# Some inequalities for generalized eigenvalues of perturbation problems on Hermitian matrices

**DOI:** 10.1186/s13660-018-1749-0

**Published:** 2018-07-03

**Authors:** Yan Hong, Dongkyu Lim, Feng Qi

**Affiliations:** 1 College of Mathematics, Inner Mongolia University for Nationalities, Tongliao, China; 2 Department of Mathematics, Sungkyunkwan University, Suwon, South Korea; 3 Institute of Mathematics, Henan Polytechnic University, Jiaozuo, China; 4 Department of Mathematics, College of Science, Tianjin Polytechnic University, Tianjin, China; 5 Institute of Fundamental and Frontier Sciences, University of Electronic Science and Technology of China, Chengdu, China

**Keywords:** 15B33, 11R52, 15A42, 15A48, 16H05, 20G20, Generalized eigenvalue, Hermitian matrix, Inequality, Perturbation problem

## Abstract

In the paper, the authors establish some inequalities for generalized eigenvalues of perturbation problems on Hermitian matrices and modify shortcomings of some known inequalities for generalized eigenvalues in the related literature.

## Introduction

Let $A,B \in\mathbb{C}^{n\times n}$ be Hermitian matrices with *B* being positive definite. We now consider a perturbation problem for $A\boldsymbol{x}= \lambda B\boldsymbol{x}$. It is known that the *n* generalized eigenvalues of the matrix pencil $\langle A,B\rangle$ are real numbers and that the generalized eigenvalues of $\langle A,B\rangle$ and the eigenvalues of $AB^{-1}$ are the same. Without loss of generality, we can line up the eigenvalues of a Hermitian matrix *A* as

$$\lambda_{1}(A)\ge\lambda_{2}(A)\ge\dotsm\ge \lambda_{n}(A) $$

and order the generalized eigenvalues of $\langle A,B\rangle$ by

$$\lambda_{1} \bigl(AB^{-1} \bigr)\ge\lambda_{2} \bigl(AB^{-1} \bigr)\ge \dotsm\ge\lambda_{n} \bigl(AB^{-1} \bigr). $$

For a standard Hermitian eigenvalue problem $A\boldsymbol{x}= \lambda \boldsymbol{x}$, Weyl’s theorem [2] is perhaps the best-known perturbation result. We denote the spectral norm of a matrix by $\|\cdot\|_{2}$ which is also called the largest singular value or the matrix 2-norm.

We now recall several known conclusions in the literature.

### Theorem 1.1

([2, Weyl’s theorem])

*Let* $A,E\in\mathbb{C}^{n\times n}$ *be Hermitian matrices*, *and let* $\widetilde{A}=A+E$ *be a perturbation of* *A*, *then*

$$\max_{1\le i\le n} \bigl\vert \lambda_{i}(A)- \lambda_{i} (\widetilde {A} ) \bigr\vert \le \Vert E \Vert _{2}. $$

### Theorem 1.2

([3])

*Let* $A,E\in\mathbb{C}^{n\times n}$ *be Hermitian matrices*, *and let* $\widetilde{A}=A+E$ *be a perturbation of* *A*, *then*

$$ \bigl\vert \lambda (\widetilde{A} )-\lambda(A) \bigr\vert \le\bigl( \Vert A \Vert _{2}+ \Vert E \Vert _{2}\bigr)^{1-1/n} \Vert E \Vert _{2}^{1/n}. $$

### Theorem 1.3

([1, 4])

*Let* $A,B \in\mathbb{C}^{n\times n}$ *be Hermitian matrices*, *and let* *B* *be a positive definite Hermitian matrix*. *Then the equalities*

$$\lambda_{i} \bigl(A B^{-1} \bigr)=\max_{\substack{S\subseteq\mathbb {C}^{n}\\ \dim S=i}} \min_{0\ne\boldsymbol{x}\in S} \biggl\{ \frac{\boldsymbol {x}^{*}A\boldsymbol{x}}{ \boldsymbol{x}^{*} B\boldsymbol{x}} \biggr\} =\min _{\substack{T\subseteq\mathbb{C}^{n}\\ \dim T=n-i+1}} \max_{0\ne\boldsymbol{x}\in T} \biggl\{ \frac{\boldsymbol{x}^{*}A\boldsymbol{x}}{\boldsymbol {x}^{*}B\boldsymbol{x}} \biggr\} $$

*hold for* $1\le i\le n$. *In particular*, *if* $B=I_{n}$, *we have*

$$ \lambda_{i}(A) =\max_{\substack{S\subseteq\mathbb{C}^{n}\\ \dim S=i}} \min _{0\ne\boldsymbol{x}\in S} \boldsymbol{x}^{*}A\boldsymbol{x} =\min _{\substack{T\subseteq\mathbb{C}^{n}\\ \dim T=n-i+1}} \max_{0\ne\boldsymbol{x}\in T} \boldsymbol{x}^{*}A\boldsymbol {x},\quad1\le i\le n. $$

### Theorem 1.4

([5, p. 336])

*Let* $A,B \in\mathbb{C}^{n\times n}$ *be Hermitian matrices and* $i,j,k,\ell,\hbar\in\mathbb{N}$ *with* $j+k-1\le i\le\ell+\hbar -n-1$. *Then*

$$ \lambda_{\ell}(A)+\lambda_{\hbar}(A)\le\lambda_{i}(A+B) \le\lambda _{j}(A)+\lambda_{k}(B). $$

*In particular*, *we have*

$$ \lambda_{i}(A)+\lambda_{n}(B)\le\lambda_{i}(A+B) \le\lambda _{i}(A)+\lambda_{1}(B). $$

Let $A,E\in\mathbb{C}^{n\times n}$ be Hermitian matrices, *B* be a positive definite Hermitian matrix,

$$\widetilde{B}=B+E,\qquad\beta_{n}=\min_{1\le i\le n} \lambda_{i}(B), \qquad \mu=\frac{ \Vert E \Vert _{2}}{\beta_{n}}=\frac{ \Vert E \Vert _{2}}{\lambda_{n}(B)}. $$

Then *μ* is a sufficient condition for *B̃* to be a Hermitian positive definite matrix.

### Theorem 1.5

([4])

*Let* $A,B,H,E \in\mathbb{C}^{n\times n}$ *be Hermitian matrices*, *B* *be a positive definite Hermitian matrix*, *and* $\widetilde{B}=B+E$. *If* $\mu =\frac{\|E\|_{2}}{\lambda_{n}(B)}<1$, *then the double inequality*

$$ (1-\mu)\lambda_{i} \bigl(AB^{-1} \bigr)+ \lambda_{n} \bigl(HB^{-1} \bigr) \le\lambda_{i} \bigl((A+H)\widetilde{B}^{-1} \bigr) \le\frac{\lambda_{i} (AB^{-1} )+\lambda_{1} (HB^{-1} )}{1-\mu} $$

*is valid for all* $1\le i\le n$.

### Theorem 1.6

([4])

*Let* $A,B,H,E \in\mathbb{C}^{n\times n}$ *be Hermitian matrices*, *B* *be a positive definite Hermitian matrix*, *and* $\widetilde{B}=B+E$. *If* $\varepsilon\triangleq\max_{1\le i\le n} |\lambda_{i} (EB^{-1} ) |<1$, *then the double inequality*

$$ (1-\varepsilon)\lambda_{i} \bigl(AB^{-1} \bigr)+ \lambda_{n} \bigl(HB^{-1} \bigr) \le\lambda_{i} \bigl((A+H)\widetilde{B}^{-1} \bigr) \le\frac{\lambda_{i} (AB^{-1} )+\lambda_{1} (HB^{-1} )}{1-\varepsilon} $$

*is valid for all* $1\le i\le n$.

### Remark 1.1

Let

$$A =\operatorname {diag}(-3, -2),\qquad B=\operatorname {diag}(3, 4),\qquad H =I_{2}, \qquad E=\operatorname {diag}(2, 1). $$

Then

$$ \lambda_{2} \bigl(HB^{-1} \bigr)+(1-\mu)\lambda_{2} \bigl(AB^{-1} \bigr)=\frac{1}{3} >0 = \lambda_{2} \bigl((A+H)\widetilde{B}^{-1} \bigr). $$

Let

$$A=\operatorname {diag}(-3,-2),\qquad B=\operatorname {diag}(3,4),\qquad H =-2I_{n},\qquad E=\operatorname {diag}(2,1). $$

Then

$$ \lambda_{1} \bigl((A+H)\widetilde{B}^{-1} \bigr) =- \frac{4}{5} >-3=\frac{\lambda_{1} (AB^{-1} )+\lambda_{1} (HB^{-1} )}{1-\varepsilon}. $$

These two examples demonstrate that Theorems 1.5 and 1.6 are not necessarily true.

In this paper, we will establish some inequalities of perturbation problems for generalized eigenvalues.

## Main results

We are now in a position to state and prove our main results in this paper.

### Theorem 2.1

*Let* $A,B,H,E \in\mathbb{C}^{n\times n}$ *be Hermitian matrices*, *B* *be a positive definite Hermitian matrix*, *and* $\widetilde{B}=B+E$. *If* $\mu =\frac{\|E\|_{2}}{\lambda_{n}(B)}<1$ *and* $i,j,k,\ell,\hbar\in\mathbb {N}$ *with* $j+k-1\le i\le\ell+\hbar-n-1$, *then*

1. *when* $\lambda_{i}(A+H)\ge0$, *we have*
  $$ \frac{\lambda_{\ell}(AB^{-1} )+\lambda_{\hbar} (HB^{-1} )}{1+\mu} \le\lambda_{i} \bigl((A+H) \widetilde{B}^{-1} \bigr) \le\frac{\lambda_{j} (AB^{-1} )+\lambda_{k} (HB^{-1} )}{1-\mu}; $$
2. *when* $\lambda_{i}(A+H)\le0$, *we have*
  $$ \frac{\lambda_{j} (AB^{-1} )+\lambda_{k} (HB^{-1} )}{1-\mu} \le\lambda_{i} \bigl((A+H) \widetilde{B}^{-1} \bigr) \le\frac{\lambda_{\ell}(AB^{-1} )+\lambda_{\hbar} (HB^{-1} )}{1+\mu}. $$

### Proof

Since $B^{-1/2}(A+H)B^{-1/2}$ is a Hermitian matrix, then there exists an orthogonal matrix $U=(\boldsymbol{u}_{1},\boldsymbol{u}_{2},\dotsc,\boldsymbol{u}_{n})\in \mathbb{C}^{n\times n}$ such that

$$ B^{-1/2}(A+H)B^{-1/2}=U^{*}\operatorname {diag}\bigl(\lambda_{1} \bigl((A+H)B^{-1} \bigr),\dotsc,\lambda_{n} \bigl((A+H)B^{-1} \bigr) \bigr)U. $$

Let

$$ T_{i}=\operatorname{Span} (\boldsymbol{u}_{i}, \boldsymbol{u}_{i+1},\dotsc,\boldsymbol{u}_{n} ),\quad1\le i\le n. $$

By virtue of Theorems 1.3 and 1.4, if $j+k-1\le i\le\ell+\hbar-n-1$, we have

2.1 $$ \begin{aligned}[b] \lambda_{i} \bigl((A+H)\widetilde{B}^{-1} \bigr) &\le\max_{0\ne\boldsymbol{x}\in T} \biggl\{ \frac{\boldsymbol{x}^{*}B^{-1/2}(A+H)B^{-1/2}\boldsymbol{x}}{ \boldsymbol{x}^{*} (I_{n}+B^{-1/2}EB^{-1/2} )\boldsymbol {x}} \biggr\} \\ &\le \textstyle\begin{cases} \frac{1}{1-\mu}\max_{0\ne\boldsymbol{x}\in T} \{\frac{\boldsymbol{x}^{*}B^{-1/2}(A+H)B^{-1/2}\boldsymbol{x}}{\boldsymbol{x}^{*}\boldsymbol{x}} \},&\lambda_{i}(A+H)\ge0; \\ \frac{1}{1+\mu}\max_{0\ne\boldsymbol{x}\in T} \{\frac{\boldsymbol{x}^{*}B^{-1/2}(A+H)B^{-1/2}\boldsymbol{x}}{\boldsymbol{x}^{*}\boldsymbol{x}} \},&\lambda_{i}(A+H)< 0 \end{cases}\displaystyle \\ &= \textstyle\begin{cases} \frac{1}{1-\mu}\lambda_{i} ((A+H)B^{-1} ),&\lambda _{i}(A+H)\ge0; \\ \frac{1}{1+\mu}\lambda_{i} ((A+H)B^{-1} ),&\lambda _{i}(A+H)< 0 \end{cases}\displaystyle \\ &\le \textstyle\begin{cases} \frac{\lambda_{j} (AB^{-1} )+\lambda_{k} (HB^{-1} )}{1-\mu},&\lambda_{i}(A+H)\ge0; \\ \frac{\lambda_{\ell}(AB^{-1} )+\lambda_{\hbar} (HB^{-1} )}{1+\mu},&\lambda_{i}(A+H)< 0. \end{cases}\displaystyle \end{aligned} $$

Similarly, we have

2.2 $$ \lambda_{i} \bigl((A+H)\widetilde{B}^{-1} \bigr) \ge \textstyle\begin{cases} \frac{\lambda_{\ell}(AB^{-1} )+\lambda_{\hbar} (HB^{-1} )}{1+\mu}, &\lambda_{i}(A+H)\ge0;\\ \frac{\lambda_{j} (AB^{-1} )+\lambda_{k} (HB^{-1} )}{1-\mu}, &\lambda_{i}(A+H)< 0. \end{cases} $$

The proof of Theorem 2.1 is complete. □

### Corollary 2.1

*Let* $A,B,H,E \in\mathbb{C}^{n\times n}$ *be Hermitian matrices*, *B* *be a positive definite Hermitian matrix*, *and* $\widetilde{B}=B+E$. *If* $\mu =\frac{\|E\|_{2}}{\lambda_{n}(B)}<1$, *then*

1. *when* $\lambda_{i}(A+H)\ge0$ *for* $1\le i\le n$,
  $$ \frac{\lambda_{i} (AB^{-1} )+\lambda_{n} (HB^{-1} )}{1+\mu} \le\lambda_{i} \bigl((A+H) \widetilde{B}^{-1} \bigr) \le\frac{\lambda_{i} (AB^{-1} )+\lambda_{1} (HB^{-1} )}{1-\mu}; $$
2. *when* $\lambda_{i}(A+H)\le0$ *for* $1\le i\le n$,
  $$ \frac{\lambda_{i} (AB^{-1} )+\lambda_{1} (HB^{-1} )}{1-\mu} \le\lambda_{i} \bigl((A+H) \widetilde{B}^{-1} \bigr) \le\frac{\lambda_{i} (AB^{-1} )+\lambda_{n} (HB^{-1} )}{1+\mu}. $$

### Corollary 2.2

*Let* $A,B,H,E \in\mathbb{C}^{n\times n}$ *be Hermitian matrices*, *B* *be a positive definite Hermitian matrix*, *and* $\widetilde{B}=B+E$. *If* $\mu =\frac{\|E\|_{2}}{\lambda_{n}(B)}<1$, *then*

1. *when* $\lambda_{i}(A+H)\ge0$ *for* $1\le i\le n$, *then*
  $$ \frac{1}{1+\mu} \biggl[\lambda_{i} \bigl(AB^{-1} \bigr)-\frac{\|H\| }{\lambda_{n}(B)} \biggr] \le\lambda_{i} \bigl((A+H) \widetilde{B}^{-1} \bigr) \le\frac{1}{1-\mu} \biggl[\lambda_{i} \bigl(AB^{-1} \bigr)+\frac{\| H\|}{\lambda_{n}(B)} \biggr]; $$
2. *when* $\lambda_{i}(A+H)\le0$ *for* $1\le i\le n$, *then*
  $$ \frac{1}{1-\mu} \biggl[\lambda_{i} \bigl(AB^{-1} \bigr)-\frac{\|H\| }{\lambda_{n}(B)} \biggr] \le\lambda_{i} \bigl((A+H) \widetilde{B}^{-1} \bigr) \le\frac{1}{1+\mu} \biggl[\lambda_{i} \bigl(AB^{-1} \bigr)+\frac{\| H\|}{\lambda_{n}(B)} \biggr]. $$

### Theorem 2.2

*Let* $A,B,H,E \in\mathbb{C}^{n\times n}$ *be Hermitian matrices*, *B* *be a positive definite Hermitian matrix*, *and* $\widetilde{B}=B+E$. *If* $\varepsilon=\max_{1\le i\le n} |\lambda_{i} (EB^{-1} ) |<1$, *then*

1. *when* $\lambda_{i}(A+H)\ge0$ *for* $1\le i\le n$,
  $$ \frac{\lambda_{i} (AB^{-1} )+\lambda_{n} (HB^{-1} )}{1+\varepsilon} \le\lambda_{i} \bigl((A+H) \widetilde{B}^{-1} \bigr) \le\frac{\lambda_{i} (AB^{-1} )+\lambda_{1} (HB^{-1} )}{1-\varepsilon}; $$
2. *when* $\lambda_{i}(A+H)\le0$ *for* $1\le i\le n$,
  $$ \frac{\lambda_{i} (AB^{-1} )+\lambda_{1} (HB^{-1} )}{1-\varepsilon} \le\lambda_{i} \bigl((A+H) \widetilde{B}^{-1} \bigr) \le\frac{\lambda_{i} (AB^{-1} )+\lambda_{n} (HB^{-1} )}{1+\varepsilon}. $$

### Proof

Using inequalities (2.1) and (2.2), we obtain the required results. The proof of Theorem 2.2 is thus complete. □

### Theorem 2.3

*Let* $A,B,H,E \in\mathbb{C}^{n\times n}$ *be Hermitian matrices*, *B* *be a positive definite Hermitian matrix*, *and* $\widetilde{B}=B+E$. *If* $\mu =\frac{\|E\|_{2}}{\lambda_{n}(B)}<1$, *then*

$$\begin{aligned} \beta_{i}(A)\lambda_{i} \bigl(AB^{-1} \bigr)+\beta_{n}(H)\lambda_{n} \bigl(HB^{-1} \bigr) &\le\lambda_{i} \bigl((A+H)\widetilde{B}^{-1} \bigr) \\ &\le\alpha_{i} (A)\lambda_{i} \bigl(AB^{-1} \bigr)+\alpha_{1}(H)\lambda _{1} \bigl(HB^{-1} \bigr)\end{aligned} $$

*for* $1\le i\le n$, *where*

$$ \alpha_{i} (A)= \textstyle\begin{cases} \frac{1}{1-\mu}, &\lambda_{i} (A)\ge0;\\ \frac{1}{1+\mu}, &\lambda_{i} (A)< 0 \end{cases}\displaystyle \quad \textit{and}\quad \beta_{i} (A)= \textstyle\begin{cases} \frac{1}{1-\mu}, &\lambda_{i}(A)< 0;\\ \frac{1}{1+\mu}, &\lambda_{i}(A)\ge0. \end{cases} $$

### Proof

Since

$$\begin{aligned} \lambda_{i} \bigl(\widetilde{B}^{-1/2} A\widetilde{B}^{-1/2} \bigr) +\lambda_{n} \bigl(\widetilde{B}^{-1/2} H \widetilde{B}^{-1/2} \bigr) &\le\lambda_{i} \bigl((A+H) \widetilde{B}^{-1} \bigr) \\ &\le\lambda_{i} \bigl(\widetilde{B}^{-1/2} A\widetilde {B}^{-1/2} \bigr) +\lambda_{1} \bigl(\widetilde{B}^{-1/2} H\widetilde{B}^{-1/2} \bigr)\end{aligned} $$

for $1\le i\le n$. From inequalities in (2.1) and (2.2), it follows that

$$\begin{gathered} \beta_{i} (A)\lambda_{i} \bigl(AB^{-1} \bigr) \le \lambda_{i} \bigl(\widetilde{B}^{-1/2}A\widetilde{B}^{-1/2} \bigr) =\lambda_{i} \bigl(A\widetilde{B}^{-1} \bigr)\le \alpha_{i} (A)\lambda _{i} \bigl(AB^{-1} \bigr), \\ \beta_{n}(H)\lambda_{n} \bigl(HB^{-1} \bigr)\le \lambda_{n} \bigl(H\widetilde{B}^{-1} \bigr),\qquad \lambda_{1} \bigl(H\widetilde{B}^{-1} \bigr)\le \alpha_{1}(H)\lambda _{1} \bigl(AB^{-1} \bigr) \end{gathered}$$

for $1\le i\le n$. The proof of Theorem 2.3 is complete. □

## References

[1] Amir-Moéz A.R. (1956). Extreme properties of eigenvalues of a Hermitian transformation and singular values of the sum and product of linear transformations. Duke Math. J..

[2] Bai Z., Demmel J., Dongarra J., Ruhe A., van der Vorst H. (2000). Templates for the Solution of Algebraic Eigenvalue Problems: A Practical Guide.

[3] Elsner L. (1985). An optimal bound for the spectral variation of two matrices. Linear Algebra Appl..

[4] Huang L. (1978). Some perturbation problems for the generalized eigenvalues. Acta Sci. Nat. Univ. Pekin..

[5] Marshall A.W., Olkin I., Arnold B.C. (2011). Inequalities: Theory of Majorization and Its Applications.

